# CD16^+^ Monocyte Subset Was Enriched and Functionally Exacerbated in Driving T-Cell Activation and B-Cell Response in Systemic Lupus Erythematosus

**DOI:** 10.3389/fimmu.2016.00512

**Published:** 2016-11-21

**Authors:** Huaqun Zhu, Fanlei Hu, Xiaolin Sun, Xiaoying Zhang, Lei Zhu, Xu Liu, Xue Li, Liling Xu, Lianjie Shi, Yuzhou Gan, Yin Su

**Affiliations:** ^1^Department of Rheumatology and Immunology, Peking University People’s Hospital, Beijing, China; ^2^Beijing Key Laboratory for Rheumatism Mechanism and Immune Diagnosis (BZ0135), Beijing, China; ^3^Peking-Tsinghua Center for Life Sciences, Beijing, China; ^4^Peking University International Hospital, Beijing, China

**Keywords:** systemic lupus erythematosus, CD16^+^ monocytes, expansion, T cell activation, B cell response

## Abstract

**Background:**

The roles that CD16^+^ monocyte subset plays in T-cell activation and B-cell response have not been well studied in systemic lupus erythematosus (SLE).

**Objective:**

The present study aimed to investigate the distribution of CD16^+^ monocyte subsets in SLE and explore their possible roles in T-cell activation and B-cell differentiation.

**Methods:**

The frequencies of monocyte subsets in the peripheral blood of healthy controls (HCs) and patients with SLE were determined by flow cytometry. Monocyte subsets were sorted and cocultured with CD4^+^ T cells and CD19^+^ B cells. Then, T and B cells were collected for different subset detection, while the supernatants were collected for immunoglobulin G, IgA, and IgM or interferon-γ and interleukin-17A detection by enzyme-linked immunosorbent assay.

**Results:**

Our results showed that CD16^+^ monocytes exhibited a proinflammatory phenotype with elevated CD80, CD86, HLA-DR, and CX3CR1 expression on the cell surface. It’s further demonstrated that CD16^+^ monocytes from patients and HCs shared different cell-surface marker profiles. The CD16^+^ subset was enriched in SLE and had an exacerbated capacity to promote CD4^+^ T cell polarization into a Th17 phenotype. Also, CD16^+^ monocytes had enhanced impacts on CD19^+^ B cells to differentiate into plasma B cells and regulatory B cells with more Ig production.

**Conclusion:**

This study demonstrated that CD16^+^ monocytes, characterized by different cell-surface marker profiles, were enriched and played a critical role in driving the pathogenic T- and B-cell responses in patients with SLE.

## Introduction

Systemic lupus erythematosus (SLE) is a chronic autoimmune disease with multiorgan damage characterized by immunological abnormalities that include deficient innate immune response and aberrant activation of autoreactive T and B cells, with subsequent production of pathogenic autoantibodies against cell nuclear components and resultant end-organ injury (1–4). Functional abnormalities in peripheral blood monocytes play a significant role in the pathogenesis of SLE, contributing to both aberrant T-cell activation and B-cell tolerance (2).

CD16 (FcγRIII) is one of the Fc receptors for immunoglobulin G (IgG) (FcγRs) (5). It is an activating FcγR that transmits activation signals through an immune-receptor tyrosine-based activation motif contained in its cytoplasmic region and mediates endocytosis and phagocytosis of immune complexes, including antibody-coated microorganisms (6). In both mice and humans, blood monocyte subsets exhibit differential surface expression of various FcγRs. For the past two decades, CD16 distinguishes human monocytes into two major subsets (CD16^+^ and CD16^−^ subsets). Subsequently, the third distinct monocyte subset driven from CD16^+^ monocytes was described, which was defined as relatively higher levels of CD14 coupled with lower CD16 expression. Therefore, human peripheral monocytes could be categorized into three subsets: the non-classical monocytes (NCM, CD14^+^CD16^++^), the intermediate monocytes (IM, CD14^++^CD16^+^), and the classical monocytes (CM, CD14^++^CD16^−^). NCM and IM are collectively addressed as CD16^+^ monocytes (7, 8).

CD16^+^ monocytes exhibit low levels of CCR2, the chemokine receptor of CCL2 also known as monocyte chemoattractant protein 1, and high levels of CX3CR1, known as the fracalkine (CX3CL1) receptor (7, 9). Gene expression reveals that non-classical and intermediate subsets are more closely related (10, 11), which is further supported by separate microarray study performed with rhesus monkeys that possess homologous monocyte subpopulations (12). CD16^+^ monocytes are considered to be pro-inflammatory, as they are better than CD16^−^ monocytes at producing the cytokines-tumor necrosis factor (TNF)-α, interleukin (IL)-6, and IL-10 in response to microbial-associated molecular patterns (13–15). The results accumulated over the past decades suggest that CD16^+^ subset is expanded in many different types of diseases, mostly under infection or inflammatory conditions (16–18). Some studies on monocyte subpopulations were performed in patients with SLE. Monocyte subsets exhibited no difference in expression of Mer tyrosine kinase between patients with SLE and normal controls (19), and IM have modulatory effects on CM (20). Mukherjee et al. showed that non-classical inflammatory monocytes increased in patients with SLE (21). Mikołajczyk et al. suggested that CD14dimCD16^+^ monocytes are associated with subclinical atherosclerosis in SLE (22).

Despite being the main driver of autoimmune diseases, the adaptive immune responses are strongly affected by innate immune cells. On the one hand, human peripheral blood monocytes are shown to act as antigen-processing cells (APCs) to activate T cells and secrete cytokines that shape T-cell differentiation in inflammatory diseases, such as rheumatoid arthritis and thrombocytopenia (23, 24). On the other hand, monocytes, which are generally regarded as precursors of tissue macrophages and dendritic cells (DCs) (25), can functionally promote B-cell differentiation and antibody secretion in patients with SLE (26, 27). A substantial literature is available on rats demonstrating the critical contribution of monocytes to autoreactive T-cell response and B-cell tolerance (28–31). Furthermore, therapies targeting monocyte-derived cytokines, such as TNFα, IL-6, or aberrant B-cell activation, have been successfully put into clinical practice, resulting in dramatically improved clinical outcomes in a number of autoimmune diseases (32–34).

To date, the specific roles of monocyte subsets have not been fully characterized with regards to T-cell activation and B-cell differentiation in SLE. In the present study, the flow cytometry analysis was first performed to compare the distribution of each subset between healthy individuals and patients with SLE. The expression of several cell-surface markers on each monocyte subset was also compared, including costimulatory receptors (CD80 and CD86), major histocompatibility complex (MHC) class II (HLA-DR), scavenger receptor (CD163), and chemokine receptors (CCR5, CX3CR1) from patients and healthy controls (HCs). Also, the role of monocyte subsets in the polarization of Th subsets was explored in patients with SLE. Furthermore, the study also investigated the capacity of SLE monocyte subsets in B-cell activation and differentiation. Understanding of the mechanisms underlying monocyte subset-mediated T-cell activation and B-cell responses in SLE might disclose novel therapeutic targets to treat this disease.

## Materials and Methods

### Patients and Controls

Sixty-two patients with SLE and 35 HCs were enrolled at the Department of Rheumatology, Peking University People’s Hospital, China, and blood samples were obtained from these subjects. All of the enrolled patients fulfilled at least four of the 2010 American College of Rheumatology revised criteria for SLE (35). Demographic, clinical, and laboratory data obtained from the medical records of the patients included age, sex, disease duration, blood cell counts, 24-h proteinuria excretion, anti-double-strand DNA (anti-dsDNA) antibody, anti-nucleosome antibody (AnuA), anti-Sm antibody, anti-SSA antibody, anti-SSB antibody, complement component 3 (C3), complement component 4 (C4), IgG, IgM, IgA, and erythrocyte sedimentation rate (ESR). Anti-dsDNA antibody, 24-h proteinuria excretion, C3, and C4 levels were used to predict SLE disease activity. All participants signed the informed consents to donate their blood samples and de-identified clinical information for research. The study was approved by the Institutional Medical Ethics Review Board of Peking University People’s Hospital.

### Cell Staining and Flow Cytometric Analysis

Monocyte subsets were detected in freshly collected blood from HC and SLE patients by flow cytometry using the following antibodies: fluorescein isothiocyanate (FITC) anti-human CD14 (Biolegend, San Diego, CA, USA, catalog: 325604), allophycocyanin (APC) anti-human CD16 (Biolegend, clone:3G8, catalog: 302012), Brilliant Violet 421 anti-human CD80 (Biolegend, catalog: 305221), Brilliant Violet 510 anti-human CD86 (Biolegend, catalog: 305431), PerCP/Cy5.5 anti-human CD163 (Biolegend, catalog: 333608), phycoerythrin (PE)/Dazzle 594 anti-human HLA-DR (Biolegend, catalog: 307653), AF700 anti-human CCR5 (Biolegend, catalog: 359116), and PE/Cyanine 7 anti-human CX3CR1 (Biolegend, catalog: 347612).

T cell subsets were detected after cocultured with monocytes using the following antibodies: PE-CF594 anti-human CD4 (BD Biosciences, San Diego, CA, USA, catalog: 562281), FITC anti-human interferon (IFN)-γ (Biolegend, catalog: 502506), PE anti-human IL-4 (eBioscience, San Diego, CA, USA, catalog: 12-7049-42), APC anti-human IL-17A (eBioscience, catalog: 17-7179-42), and AF 647 anti-human Foxp 3 (Biolegend, catalog: 320114) Abs.

B cell subsets were detected after cocultured with monocytes using the following antibodies: APC-Cyanine 7 anti-human CD19 (Biolegend, catalog: 302218), FITC anti-human IgD (Biolegend, catalog: 348206), PE anti-human CD24 (eBioscience, catalog: 12-0247-42), PE-Cyanine 7 anti-human CD20 (eBioscience, catalog: 25-0209-42), and APC anti-human CD27 (eBioscience, catalog: 17-0279-42) mAbs were used.

**Figure 1:**
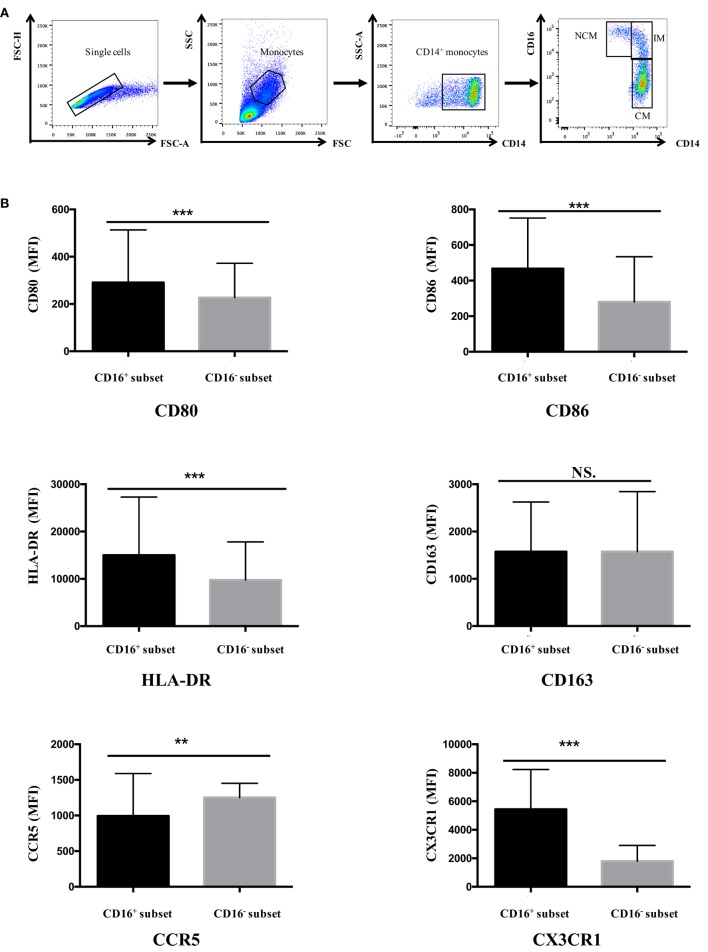
**CD16^+^ monocytes exhibited different cell-surface marker expression compared with CD16^−^ monocytes**. All samples were collected from fresh blood. **(A)** Representative dot plots in peripheral blood mononuclear cells showed the gating strategy used based on CD14 and CD16 expression. CD16^+^ monocytes could be divided into NCM and IM. **(B)** CD16^+^ and CD16^−^ monocyte subsets showed distinctive patterns of cell-surface receptor expression in HCs. NCM, non-classical monocytes; IM, intermediate monocytes; CM, classical monocytes. Data were expressed as mean ± SD and analyzed by non-parametric paired *t* test. **P < 0.01, ***P < 0.001; NS, no significance.

Gating of the single cells in a forward scatter (FSC)-A and FSC-H plot and settings of gates for detecting monocyte subsets were shown in Figure 1A. Data were analyzed with FlowJo vX0.7 (Becton Dickinson).

### Cell Isolation

Venous blood samples (12 mL) were obtained from all subjects. Human peripheral blood mononuclear cells were isolated from the blood of healthy individuals and patients with SLE using a Ficoll gradient centrifugation protocol. Monocyte subsets were purified by flow cytometry (BD FACSAria II) based on CD14 and CD16 staining. B and T cells were also isolated by flow cytometry. Figure S1 in Supplementary Material demonstrates the gating strategies about the enrichment and purification of monocyte subsets, B cells, and T cells during cell sorting. Sorted cells were further assessed by flow cytometric analysis with purity >95, >95, and >95% for monocyte subsets, B cells, and T cells, respectively (Figure S2 in Supplementary Material).

### Cell Coculture

Fresh SLE blood or healthy control buffy coat samples from blood bank were collected for cell coculture assays. The monocytes were incubated in RPMI 1640 supplemented with 10% heat-inactivated fetal calf serum, 100 U/mL penicillin, and 100 μg/mL streptomycin. To evaluate the effects of monocyte subsets on T-cell activation, each monocyte subset was cocultured with autologous CD4^+^ T cells (ratio 1:5) in the presence of anti-CD3 (1 μg/mL) (eBioscience, catalog: 16-0037) and anti-CD 28 (1 μg/mL) (eBioscience, catalog: 16-0289) antibodies (36) and macrophage colony-stimulating factor (M-CSF) (50 ng/mL) (Peprotech, Rocky Hill, CT, USA, catalog: 300-35). After 5 days, T cells were stimulated for 5 h with phorbol 12-myristate 13-acetate (PMA) (50 ng/mL), ionomycin (1 μg/mL), and brefeldin A (BFA) (10 μg/mL) (Lian ke, Hanzhou, China, catalog: CS0001, CS0002, and CS0003). Then, T cells were assessed by staining with anti-IFN-γ (Th1), anti-IL-4 (Th2), and anti-17A (Th17) antibodies. Regulatory T cells (Treg, Foxp3^+^CD4^+^) were monitored by intracellular anti-Foxp3 staining. Coculture supernatants were collected for IFN-γ and IL-17A measurement.

To explore the roles of monocyte subsets on B-cell response, each monocyte subset was cocultured with CD19^+^ B cells from the same donors (ratio 1:2.5) in the presence of anti-CD40 antibody (37) (3 μg/mL) (eBioscience, catalog:16-0409) and M-CSF (50 ng/mL) (Peprotech, catalog: 300-35) for 3 days, after which B cells were assessed for the following subpopulations: memory B cells (MBs, CD19^+^IgD^−^CD27^+^), plasma B cells (PBs, CD19^+^CD20^−^CD27^+^), and regulatory B cells (Bregs, CD19^+^CD24^+^CD27^+^). Coculture supernatants were also harvested for Ig measurement.

### T-Cell Proliferation Assay

For labeling isolated T cells, the cells were incubated for 10 min at 37°C in 5 mM carboxyfluorescein succinimidyl ester (CFSE) (Invitrogen, Carlsbad, CA, USA, catalog: C34554) in phosphate-buffered saline/0.1% bovine serum albumin (BSA/0.1% PBS). Labeling was stopped by adding five volumes of ice-cold RPMI 1640 to the cells. The cells were washed three times before use. Labeled T cells were cocultured with each monocyte subset for 60 h, as described above. T-cell proliferation was defined as CFSE low population indicative of cell division as detected by flow cytometry.

### Enzyme-Linked Immunosorbent Assay

Commercially available enzyme-linked immunosorbent assay (ELISA) kits used for measuring IFN-γ, IL-17A, or Ig levels in the supernatants were as follows: IFN-γ ELISA kit from R&D systems (Minneapolis, MN, USA, catalog: DY285); IL-17A ELISA kit from Neobioscience Technology Co., Ltd. (Shenzhen, China, catalog: EHC 170); and IgG, IgA, and IgM ELISA kits from eBioscience (San Diego, CA, USA, catalog: 88-50550, 88-50600, and 88-50620).

### Statistical Analysis

All values were expressed as means ± SD. Data were analyzed for significance using the Statistical Package for Social Sciences version 23.0 (SPSS, Chicago, IL, USA) by non-parametric paired *t* test and Mann–Whitney *U* test. Spearman’s correlation coefficient (*r*) was applied to determine the correlation between two numerical data. A *P*-value <0.05 was considered statistically significant.

## Results

### CD16^+^ Monocyte Subset Demonstrated Different Phenotypes from CD16^−^ CM

Human peripheral blood monocytes could be categorized into CD16^+^ monocytes and CD16^−^ monocytes based on differential CD16 expression. The expression of several surface markers, including costimulatory receptors (CD80 and CD86), MHC class II (HLA-DR), scavenger receptor (CD163), and chemokine receptors (CCR5and CX3CR1), was analyzed to determine the phenotypic difference between CD16^+^ monocyte subset and CD16^−^ CM in HC donors.

CD16^+^ monocyte subset demonstrated higher expression of CD80, CD86, and HLA-DR compared with CD16^−^ CM in control donors (*P* < 0.001). No difference in CD163 expression was detected between CD16^+^ and CD16^−^ monocytes (*P* > 0.05). CCR5 expression was significantly lower in CD16^+^ subset compared with CD16^−^ monocytes (*P* < 0.01). Also, the chemokine receptor CX3CR1 was significantly more expressed on CD16^+^ monocytes than on CD16^−^ monocytes (*P* < 0.001) (Figure 1B).

### CD16^+^ Monocyte Subset Was Enriched in Patients with SLE

Skewed monocyte subsets have been reported in several autoimmune inflammatory diseases such as rheumatoid arthritis (17). The distribution of monocytes was measured in a cohort of 62 patients with SLE and 35 age-matched HCs to investigate whether this change also existed in SLE. The patients with SLE consisted of 57 female and 5 male subjects, and the median disease duration was 60 months with a range of 3–432 months.

**Figure 2:**
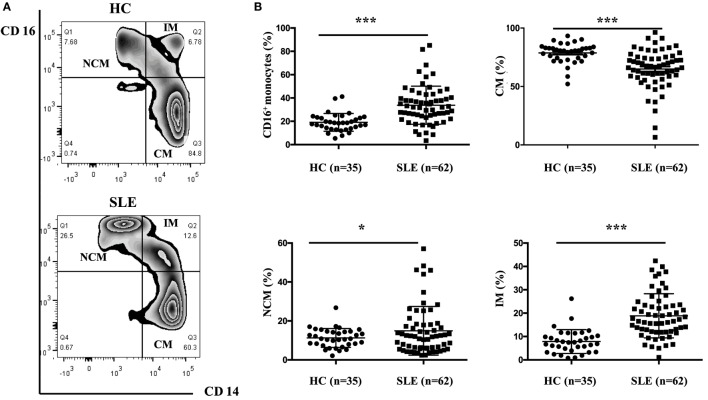
**CD16^+^ monocytes as well as their subsets (NCM and IM) were expanded in patients with SLE**. All samples were collected from fresh blood. **(A)** Representative flow charts showed the percentage of NCM, IM, and CM from a patient with SLE and a healthy control (HC). **(B)** Frequencies of NCM, IM, CM, and CD16^+^ subset were compared between 62 patients with SLE and 35 age-matched HCs. NCM, non-classical monocytes (CD14^+^CD16^++^); IM, intermediate monocytes (CD14^++^CD16^+^); CM, classical monocytes (CD14^++^CD16^−^). Data were expressed as mean ± SD and analyzed by Mann–Whitney *U* test. *P < 0.05, ***P < 0.001; NS, no significance.

The frequencies of CD16^+^ monocyte subset were significantly elevated in patients with SLE than in healthy individuals (SLE: 33.78 ± 16.19% vs. HC: 19.1 ± 7.49%, *P* < 0.001). Patients with SLE exhibited a significantly higher percentage of NCM (SLE: 14.92 ± 12.51% vs. HC: 11.27 ± 4.83%, *P* < 0.05) and IM (SLE: 18.86 ± 9.44% vs. HC: 7.84 ± 5.09%, *P* < 0.001), with obviously reduced frequencies of CM (SLE: 64.96 ± 16.87% vs. HC: 78.72 ± 8.50%, *P* < 0.001) (Figure 2) when compared with control donors.

In patients with SLE, the frequencies of CD16^+^ monocytes were positively correlated with serum IgA concentrations (*r* = 0.267; *P* < 0.05) and anti-dsDNA antibody levels (*r* = 0.349; *P* < 0.01), which implicated that patients with elevated frequencies of CD16^+^ monocytes would be at a high risk of Ig production or autoantibody development. On the contrary, the percentage of CD16^−^ subsets was negatively correlated with serum IgA (*r* = −0.26; *P* < 0.05) and anti-dsDNA antibody levels (*r* = −0.345; *P* < 0.01). The frequencies of NCM subset were correlated with anti-dsDNA antibody levels (*r* = 0.302; *P* < 0.05), while the frequencies of IM subset were correlated with serum IgA levels (*r* = 0.287; *P* < 0.05). Other laboratory data, including IgG, IgM, C3 level, C4 level, AnuA, ESR, and 24-h proteinuria excretion, did not show significant correlation with monocyte subsets (Table 1). The frequencies of monocyte subsets were further compared between patients with and without particular antibodies, including AnuA, anti-Sm Ab, anti-SSA Ab, and anti-SSB Ab. Detailed analysis of such autoantibodies in the patient cohort revealed no predictive factors for the expansion of CD16^+^ monocytes (Table S1 in Supplementary Material). The correlation analysis revealed slightly weak correlation between monocyte subsets and serum anti-dsDNA Abs or IgA levles, indicating that monocyte subset might contribute to B cell response in SLE.

**Table 1:** **Correlations between each monocyte subset frequencies and clinical parameters in SLE**.

Clinical parameters	CD16^+^ subset (%)		NCM (%)		IM (%)		CM (%)	
	Spearman’s *r*	*P*	Spearman’s *r*	*P*	Spearman’s *r*	*P*	Spearman’s *r*	*P*
IgA	0.267*	0.038	0.012	0.925	0.287*	0.025	−0.26*	0.043
IgG	0.061	0.642	0.045	0.733	0.095	0.472	−0.055	0.678
IgM	−0.095	0.472	−0.15	0.252	−0.1	0.466	0.09	0.494
C3	0.092	0.481	0.116	0.372	0.06	0.648	−0.071	0.584
C4	0.061	0.642	0.004	0.979	0.185	0.156	−0.031	0.816
Anti-dsDNA Ab	0.349**	0.006	0.302*	0.018	0.129	0.32	−0.345**	0.006
AnuA	0.079	0.533	0.093	0.484	0.055	0.682	−0.098	0.462
ESR	−0.171	0.187	−0.091	0.484	−0.14	0.281	0.164	0.206
24-h proteinuria	0.059	0.681	−0.051	0.722	−0.228	0.107	0.188	0.187

*P < 0.05.

**P < 0.01.

*SLE, systemic lupus erythematosus; Anti-dsDNA Ab, anti-double-strand DNA antibody; AnuA, anti-nucleosome antibody; C3, complement 3; C4, complement 4, ESR, erythrocyte sedimentation rate. Spearman’s correlation coefficient (*r*) was applied to detect correlation between two numerical data*.

### CD16^+^ Monocytes in Patients with SLE Shared Different Cell-Surface Marker Profiles from CD16^+^ Monocytes in HCs

**Figure 3:**
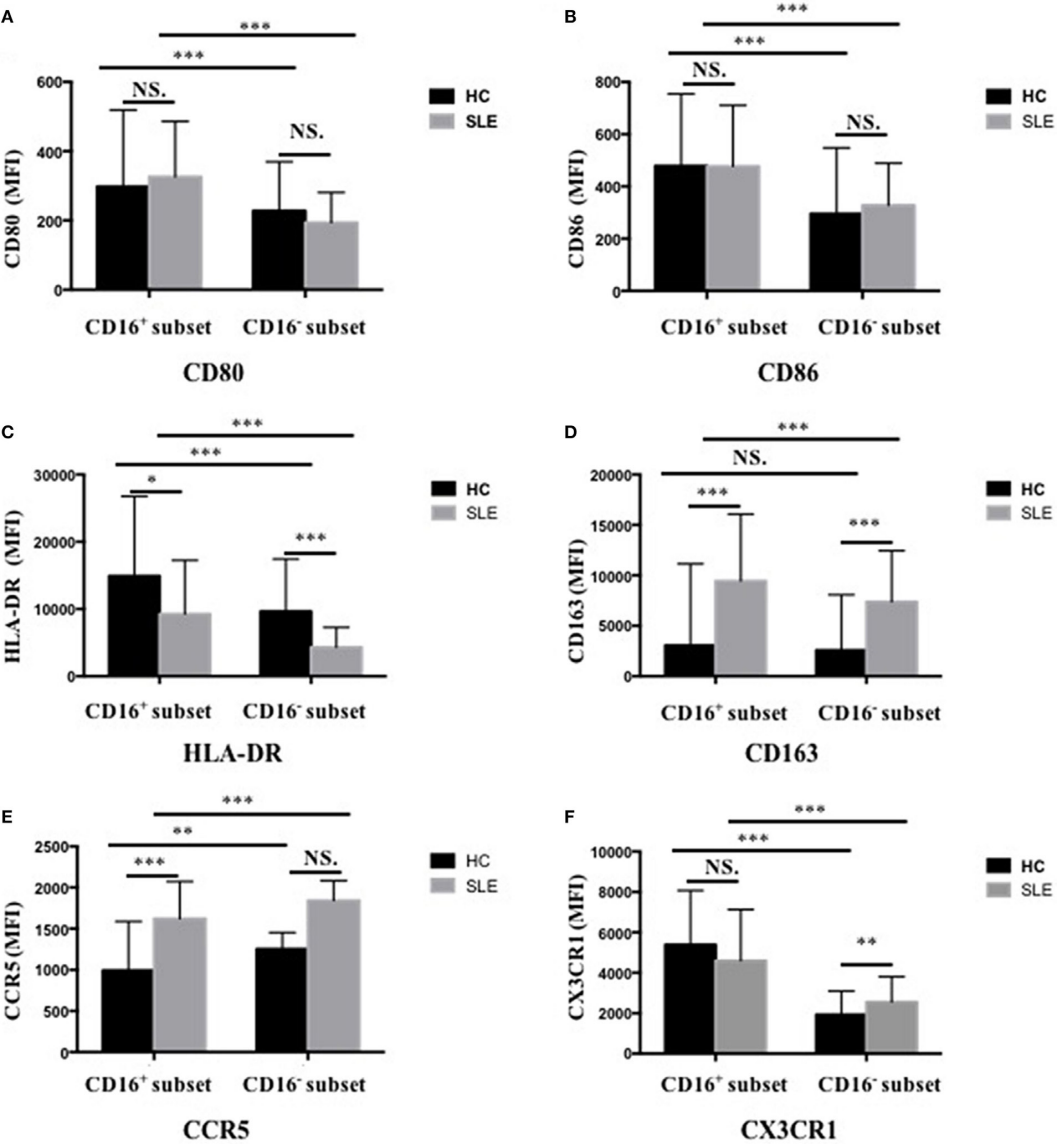
**CD16^+^ monocytes as well as CD16^−^ monocytes from patients with SLE and HCs shared different phenotypes regarding cell-surface markers, including CD80 (A), CD86 (B), HLA-DR (C), CD163 (D), CCR5 (E), and CX3CR1 (F)**. MFI, median fluorescence intensity. All samples were collected from fresh blood. Data were expressed as mean ± SD and analyzed by non-parametric paired *t* test and Mann–Whitney *U* test. *P < 0.05, **P < 0.01, ***P < 0.001; NS, no significance.

CD16^+^ monocytes from patients with SLE were characterized by lower expression of HLA-DR (*P* < 0.05) but higher CD163 and CCR5 (*P* < 0.001) expression compared with the monocytes from control donors (*P* < 0.001) (Figures 3C–E; Table S2 in Supplementary Material). CD16^−^ monocytes from patients with SLE also expressed less HLA-DR but more CD163 compared with those from HCs (*P* < 0.001) (Figures 3C,D; Table S2 in Supplementary Material). Specially, CX3CR1 expression on CD16^−^ monocytes was elevated in patients with SLE (*P* < 0.01) (Figure 3F; Table S2 in Supplementary Material). No difference in CD80 and CD86 expression on both CD16^+^ monocytes and CD16^−^ monocytes was detected between patients with SLE and HCs (Figures 3A,B; Table S2 in Supplementary Material). In SLE patients, CD16^+^ monocytes also shared different phenotypes from CD16^−^ monocytes (Figure 3). CD163 is an anti-inflammatory marker, whereas HLA-DR is a proinflammatory marker. These data demonstrated the downregulation of proinflammatory surface markers but the upregulation of anti-inflammatory markers in SLE, which was different from the presumptive results. This discrepancy might be due to a negative feedback existing to maintain monocyte homeostasis in SLE.

### CD16^+^ Monocytes Perpetuated T-Cell-Mediated Inflammation in SLE

Purified monocyte subsets with CD4^+^ T cells from the same donors were cultured in the presence of anti-CD3 and anti-CD28 antibodies and M-CSF to evaluate the effects of CD16^+^ and CD16^−^ monocytes on T-cell function. Anti-CD3 and anti-CD28 antibodies were added for supporting T-cell activation, while M-CSF was added for monocyte survival maintenance.

#### CD16^+^ Monocytes Were More Functionally Effective on Promoting T-Cell Responses Compared with CD16^−^ Monocytes in HCs

**Figure 4:**
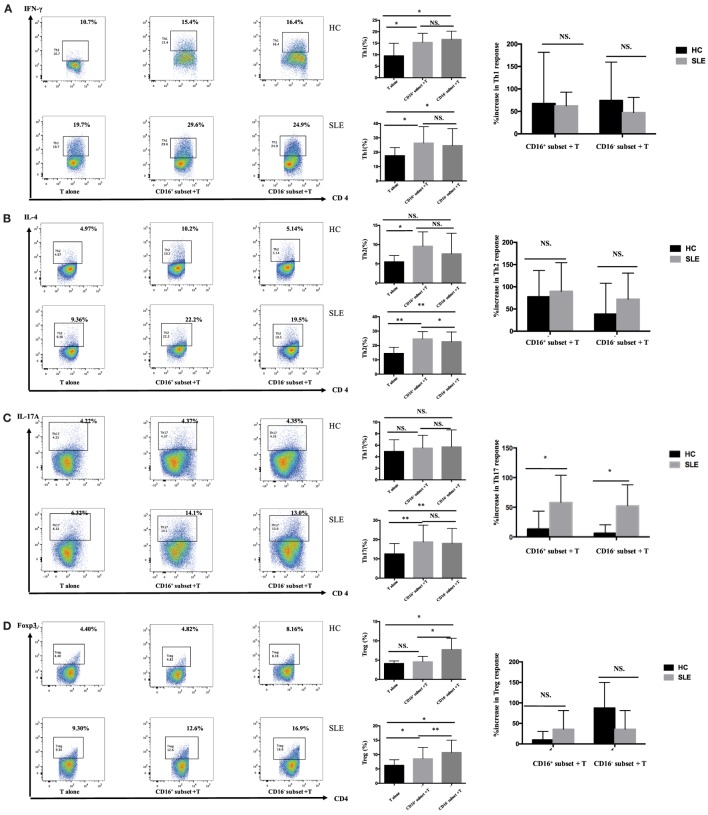
**CD16^+^ monocytes promoted T-cell-mediated inflammation in SLE**. CD16^+^ or CD16^−^ monocytes were cocultured with CD4^+^ T cells isolated from freshly collected SLE blood or blood bank collected HC blood buffy coat for 5 days in the presence of anti-CD3 (1 μg/mL) and anti-CD28 (1 μg/mL) antibodies and M-CSF (50 ng/mL). Intracellular IFN-γ, IL-4, and IL-17A expression was detected by flow cytometry after PMA (50 ng/mL), ionomycin (1 μg/mL), and BFA (10 μg/mL) stimulation for 5 h on day 5. The percentage of Treg (CD4^+^Foxp3^+^) was also analyzed. Representative pseudocolor dots depicted Th1 **(A)**, Th2 **(B)**, Th17 **(C)**, and Treg **(D)** frequencies in CD4^+^ T cells after coculture with each monocyte subset from one patient and one control donor. The proportion of Th1, Th2, Th17, and Treg cells was calculated after coculture of CD4^+^ T cells with each monocyte. The percentage increases in T-cell subsets in cocultures of monocytes and T cells compared with CD4^+^ T cells cultured alone were compared between 7 healthy individuals and 10 patients with SLE. Data were expressed as mean ± SD and analyzed by non-parametric paired *t* test and Mann–Whitney *U* test. *P < 0.05, **P < 0.01; NS, no significance.

CD4^+^ T cells demonstrated stronger Th1 (IFN-γ^+^CD4^+^) and Th2 (IL-4^+^CD4^+^) cell responses in the presence of CD16^+^ monocytes (*P* < 0.05) (Figures 4A,B). CD4^+^ T cells cocultured with CD16^−^ monocytes also contained higher numbers of Th1 cells than did CD4^+^ T cells cultured alone (*P* < 0.05) (Figure 4A). Nevertheless, CD16^−^ monocytes did not efficiently promote Th2 activation in HCs (Figure 4B). No difference in the induction of Th17-cell activation was observed (Figure 4C). Compared with the CD16^−^ subset, CD16^+^ monocytes had no effect on promoting Treg (Foxp3^+^ CD4^+^) differentiation in HCs (Figure 4D).

#### CD16^+^ Monocytes Enhanced Th17-Cell Differentiation in SLE

In patients with SLE, the differentiation of CD4^+^ T cells into Th1, Th2, and Th17 cells increased in the presence of either CD16^+^ or CD16^−^ monocytes (Figures 4A–C). Specially, CD16^+^ monocytes induced more Th2 responses compared with CD16^−^ monocytes in patients with SLE (*P* < 0.05) (Figure 4B). CD16^+^ and CD16^−^ monocytes from SLE patients were both potent at promoting CD4^+^ T cells to differentiate into Treg cells, although CD4^+^ T cells cocultured with CD16^+^ monocytes demonstrated weak effects on Treg responses than did CD4^+^ T cells cocultured with CD16^−^ monocytes (*P* < 0.01) (Figure 4D).

The two monocyte subsets, particularly CD16^+^ monocytes, induced a significant increase in Th17 cells in patients with SLE compared with HCs (*P* < 0.05) (Figure 4C). However, no difference was found in the induction of Th1, Th2, and Treg cells by CD16^+^ monocytes and CD16^−^ monocytes between patients with SLE and HCs, respectively (Figures 4A,B,D).

#### CD16^+^ Monocytes Induced T Cell Proinflammatory Cytokine Production in SLE

**Figure 5:**
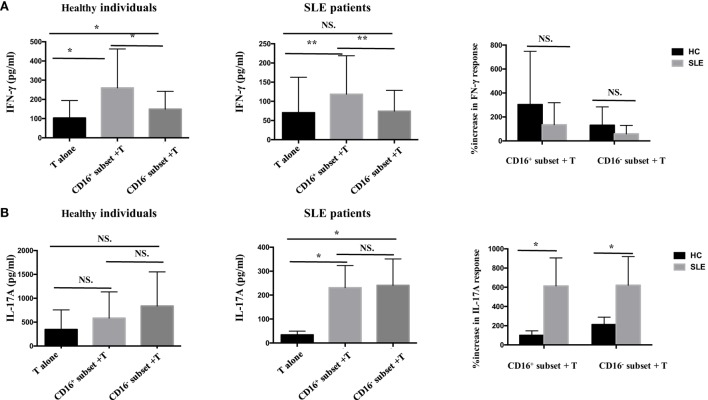
**CD16^+^ monocytes promoted T-cell-mediated cytokine secretion in SLE**. CD16^+^ or CD16^−^ monocytes were cocultured with CD4^+^ T cells isolated from freshly collected SLE blood or blood bank collected HC blood buffy coat for 5 days in the presence of anti-CD3 (1 μg/mL) and anti-CD28 (1 μg/mL) antibodies and M-CSF (50 ng/mL). The concentrations of IFN-γ and IL-17A in the supernatants were measured by ELISA. IFN-γ **(A)** and Th17A **(B)** levels were compared between different groups in HCs and patients with SLE. Data were expressed as mean ± SD and analyzed by non-parametric paired *t* test and Mann–Whitney *U* test. *P < 0.05, **P < 0.01; NS, no significance.

Next, whether CD16^+^ monocytes could influence the secretion of IFN-γ and IL-17A by T cells was assessed. Both CD16^+^ monocytes from patients with SLE and HCs upregulated IFN-γ secretion. However, no difference was found in the secretion of IFN-γ induced by CD16^+^ monocytes and CD16^−^ monocytes between patients with SLE and HCs, respectively (Figure 5A). The two monocyte subsets, particularly CD16^+^ monocytes, induced a significant increase in IL-17A secretion in patients with SLE compared with HCs (Figure 5B), which was consistent with the flow cytometry data. Of note, the baseline concentrations of IFN- γ and IL-17A in HCs were higher than SLE, probably due to the *in vitro* operation-induced slight activation (collected from buffy coat).

#### CD16^+^ Monocytes Promoted T-Cell Proliferation in SLE

**Figure 6:**
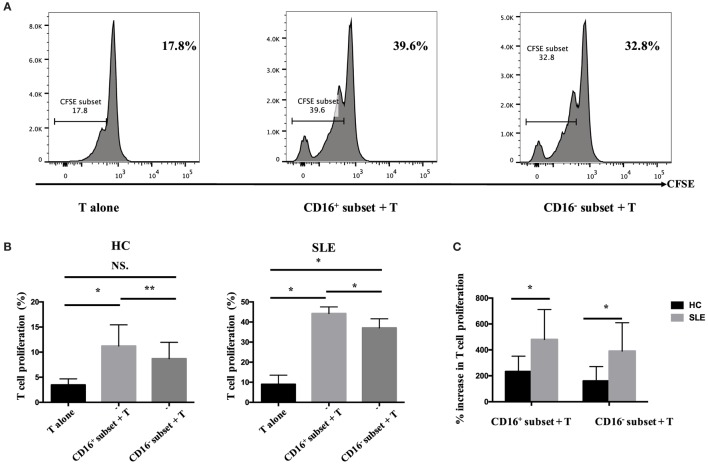
**CD16^+^ monocytes promoted T-cell proliferation in SLE**. CFSE-labeled CD4^+^ T cells isolated from freshly collected SLE blood or blood bank collected HC blood buffy coat were cocultured with CD16^+^ and CD16^−^ monocytes for 60 h in the presence of anti-CD3 (1 μg/mL) and anti-CD 28 (1 μg/mL) antibodies and M-CSF (50 ng/mL). CFSE histograms depicted the number of events (*y*-axis) and the fluorescence intensity (*x*-axis), with proliferating cells displaying a progressive loss in fluorescence intensity following cell division, indicative of proliferating cells. **(A)** Representative histogram plots from a patient with SLE displayed the frequencies of CFSE^low^ T cells in monocyte/T-cell cocultures and T cells cultured alone. **(B)** Percentages of CFSE^low^ T cells in CD4^+^ T cells after coculture with each monocyte subset from five healthy donors and six patients with SLE. **(C)** The percentage increase in CFSE^low^ T cells in cocultures of monocytes and T cells compared with CD4^+^ T cells cultured alone was compared between five healthy individuals and six patients with SLE. Data were expressed as mean ± SD and analyzed by non-parametric paired *t* test and Mann–Whitney *U* test. *P < 0.05, **P < 0.01; NS, no significance.

The percentage of CFSE^low^CD4^+^ T cells in T cells cocultured with CD16^+^ monocytes was significantly higher compared with those in T cells cocultured with CD16^−^ monocytes or T cells cultured alone (*P* < 0.01 and *P* < 0.05), indicating that CD16^+^ monocytes were the predominant monocyte subset stimulating T-cell proliferation in HCs. Similarly, CD16^+^ monocytes from SLE patients also induced significant CD4^+^ T-cell proliferation (*P* < 0.05). Importantly, CD16^−^ subset from patients with SLE also promoted CD4^+^ T-cell proliferation. However, CD16^+^ monocyte subset was more efficient than CD16^−^ subset at promoting CD4^+^ T-cell proliferation in SLE (*P* < 0.05) (Figures 6A,B). T-cell proliferation was enhanced when cocultured with both SLE monocyte subsets, particularly CD16^+^ monocytes, suggesting the potential proinflammatory phenotype of monocyte subsets in SLE when compared with HCs (Figure 6C).

### CD16^+^ Monocyte Subset Exacerbated B-Cell Activation in SLE

To test the effects of CD16^+^ and CD16^−^ monocytes on B-cell activation, purified total B cells (CD19^+^) were cocultured with or without autologous monocyte subsets in the presence of anti-CD40 antibody and M-CSF. Anti-CD40 antibody and M-CSF supported B-cell and monocyte survival, respectively. Then, the levels of IgG, IgA, and IgM were detected in the coculture supernatants to explore further whether each monocyte subset could impact the total antibody response.

#### CD16^+^ Monocytes Induced CD19^+^ B Cells to Differentiate into MBs and PBs but Inhibited the Generation of Breg Cells in HCs

**Figure 7:**
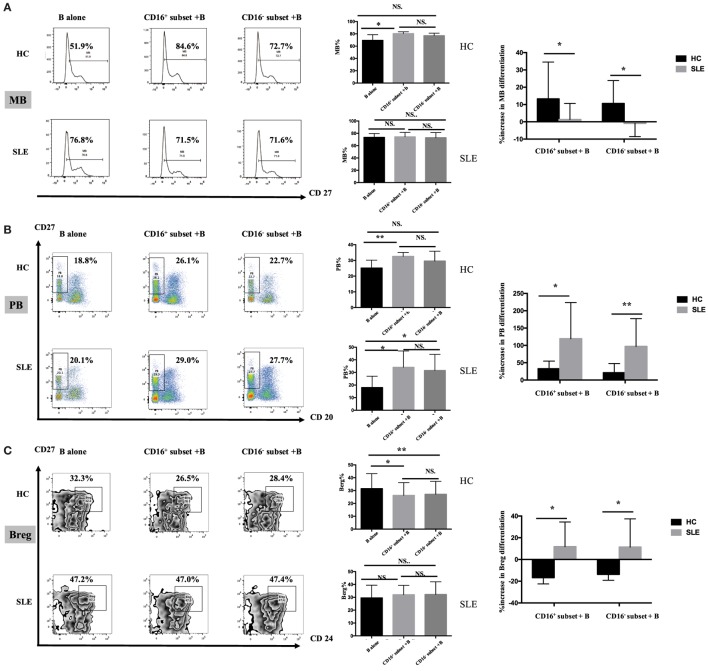
**CD16^+^ monocyte subset exacerbated B-cell activation in SLE**. SLE CD16^+^ monocytes had an enhanced capacity on PB and Breg differentiation but attenuated MB development. Monocyte subsets were cocultured with CD19^+^ B cells isolated from freshly collected SLE blood or blood bank collected HC blood buffy coat for 3 days in the presence anti-CD40 antibody (3 μg/mL) and M-CSF (50 ng/mL). The expression of CD20 and CD27 as well as CD24 was assessed. **(A)** Representative histogram plots from HCs and patients with SLE displayed the frequencies of MB (CD27^+^CD19^+^) cells in monocyte/B-cell cocultures. Graphs showed the cumulative MB frequencies in cocultures of monocytes/B cells and CD19^+^ B cells cultured alone from eight healthy individuals and seven patients with SLE. The difference in the percentage increase in MB cells in cocultures of monocytes and B cells compared with CD19^+^ B cells cultured alone was compared between HCs and SLEs. **(B)** Representative dot plots from HCs and patients with SLE demonstrated the frequencies of PB (CD20^−^CD27^+^CD19^+^) cells in monocyte/B-cell cocultures. Graphs showed the cumulative frequencies of PBs in cocultures of monocytes/B cells and CD19^+^ B cells cultured alone from eight healthy individuals and seven patients. Percentage increase in PBs in cocultures of monocytes and B cells compared with CD19^+^ B cells cultured alone was identified between HCs and patients with SLE. **(C)** Representative contour plots from HCs and patients with SLE showed the frequencies of Breg (CD24^+^CD27^+^CD19^+^) cells in monocyte/B-cell cocultures. Graphs displayed the cumulative frequency of Bregs in cocultures of monocytes/B cells and CD19^+^ B cells cultured alone from eight healthy individuals and five patients with SLE. Percentage increase in Bregs in cocultures of monocytes and B cells compared with CD19^+^ B cells cultured alone was compared between HCs and patients with SLE. MBs, memory B cells; PBs, plasma B cells; Bregs, regulatory B cells. Data were expressed as mean ± SD and analyzed by non-parametric paired *t* test and Mann–Whitney *U* test. *P < 0.05, **P < 0.01; NS, no significance.

CD16^+^ monocytes promoted CD19^+^ B cells to differentiate into MBs and PBs in HCs (Figures 7A,B). However, CD19^+^ B cells cocultured with CD16^−^ monocytes did not exhibit significantly increased frequencies of MBs and PBs. Both CD16^+^ and CD16^−^ subsets suppressed CD19^+^ B cells to differentiate into Breg cells (Figure 7C). CD19^+^ B cells demonstrated more Breg differentiation inhibition, although not significant, in the presence of CD16^+^ monocytes compared with CD16^−^ subset (Figure 7C).

#### Both PB Activation and Breg Differentiation Induced by CD16^+^ Monocytes Were Exacerbated in Patients with SLE

Both CD16^+^ and CD16^−^ monocytes had no effects on MB responses (Figure 7A), while both monocyte subsets, particularly CD16^+^ monocytes, were effective at inducing B cells to differentiate into PBs in SLE (Figure 7B). In SLE, both CD16^+^ and CD16^−^ monocytes slightly promoted Breg differentiation (Figure 7C).

MB activation induced by CD16^+^ monocytes was significantly attenuated in patients with SLE compared with HCs (*P* < 0.05), and MB activation induced by CD16^−^ SLE subset was even inhibited (*P* < 0.05) (Figure 7A). In contrast, PB activation induced by CD16^+^ monocytes and CD16^−^ subset was enhanced in SLE (Figure 7B). The phenomenon was also observed in Breg cell differentiation (Figure 7C). In patients with SLE, both CD16^+^ and CD16^−^ subsets promoted Breg cell responses, which were different from the inhibitory roles of CD16^+^ and CD16^−^ monocytes on Breg differentiation in HCs (Figure 7C).

#### CD16^+^ Monocytes Were More Effective in Stimulating B-Cell IgG Secretion in SLE

**Figure 8:**
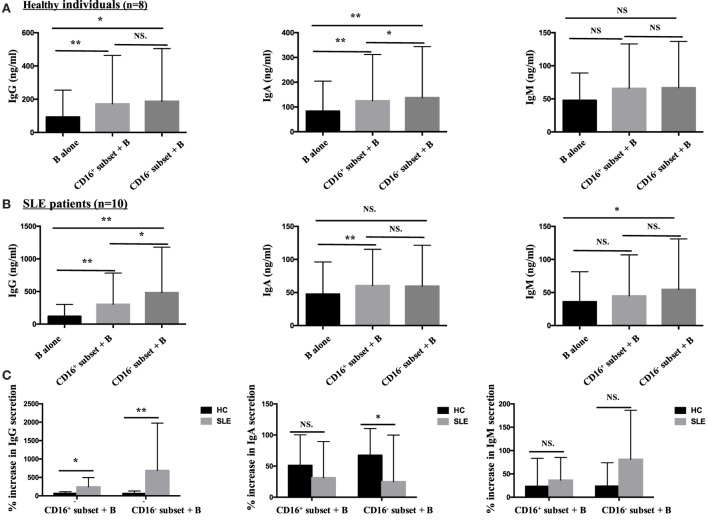
**CD16^+^ monocytes were more effective in stimulating B-cell IgG secretion in patients with SLE**. CD16^+^ or CD16^−^ monocytes were cocultured with CD19^+^ B cells isolated from freshly collected SLE blood or blood bank collected HC blood buffy coat for 3 days in the presence of anti-CD40 antibody (3 μg/mL) and M-CSF (50 ng/mL). The total Ig was assayed by ELISA. **(A)** Bar graphs showed the Ig (IgG, IgA, and IgM) levels in the supernatant of monocyte/B-cell cocultures and CD19^+^ B cells cultured alone from eight healthy individuals. **(B)** Bar graphs demonstrated the Ig (IgG, IgA, and IgM) levels in the supernatant of monocyte/B-cell cocultures and CD19^+^ B cells cultured alone from 10 patients with SLE. **(C)** The Ig increase in cocultures of monocytes and B cells compared with CD19^+^ B cells cultured alone was compared between eight healthy individuals and 10 patients with SLE. Data were expressed as mean ± SD and analyzed by non-parametric paired *t* test and Mann–Whitney *U* test. *P < 0.05, **P < 0.01; NS, no significance.

Both CD16^+^ monocytes and CD16^−^ monocytes efficiently induced CD19^+^ B cells to secrete IgG and IgA in HCs. In particular, CD16^+^ subset was less efficient than CD16^−^ monocytes at promoting IgA production. Both CD16^+^ and CD16^−^ subsets had no effect on IgM response (Figure 8A).

Both CD16^+^ and CD16^−^ monocytes were potent at inducing CD19^+^ B cells to produce IgG in SLE. CD19^+^ B cells cocultured with CD16^+^ monocytes from SLE patients secreted elevated levels of IgA. On the contrary, CD19^+^ B cells cocultured with CD16^−^ monocytes exhibited increased production of IgM in SLE (Figure 8B). Compared with HCs, IgG responses induced by CD19^+^ B cells in the presence of CD16^+^ (*P* < 0.05) and CD16^−^ (*P* < 0.01) subsets were significantly exacerbated in patients with SLE. CD16^−^ monocytes from patients with SLE induced less IgA secretion compared with CD16^−^ monocytes from HCs (*P* < 0.05). No difference in IgM response induced by CD16^+^ and CD16^−^ subsets between patients with SLE and HCs was observed (Figure 8C). Of note, the baseline concentrations of Ig levels in HCs were a bit higher than SLE, probably due to the *in vitro* operation-induced slight activation (collected from buffy coat).

## Discussion

This study showed that an enrichment of CD16^+^ monocytes in the peripheral blood of patients with SLE is associated with serum autoantibody production and that CD16^+^ monocytes exhibited a proinflammatory phenotype with high CD80, CD86, HLA-DR, and CX3CR1 expression. In SLE, CD16^+^ monocyte subset induced both Th1/Th2 cell expansion and promoted Treg development and had an enhanced capacity to promote T-cell proliferation and differentiation into a Th17 phenotype. The study demonstrated for the first time that CD16^+^ monocytes from patients with SLE could efficiently drive B-cell responses, with exacerbated impacts on PB and Breg differentiation as well as IgG production but attenuated effects on the generation of MB cells.

This study showed that the frequencies of CD16^+^ subset increased, while CD16^−^ monocytes decreased in patients with SLE. Further analysis showed that the proportions of non-classical and IM were higher in SLE than their healthy counterparts, which was consistent with the findings of Mukherjee (21). This observation was also consistent with the data showing that CD16^+^ monocyte subsets are enriched in some autoimmune diseases and may be involved in the induction of inflammatory immune response (38–41). The possible explanation of monocyte alteration is that the *in vivo* cytokine and hormone environments in SLE may lead to the conversion of CD16^−^ monocytes into CD16^+^ monocytes (20). It was shown that CD16^+^ monocytes were the producers of proinflammatory cytokines, including TNFα, IL-1, and IL-6 (13–16, 42). Mikołajczyk et al. demonstrated that CD14dimCD16^+^ monocytes might be an important subpopulation of proinflammatory monocytes related to increased development of atherosclerosis in SLE (22). The elevated surface expression of CD80, CD86, HLA-DR, and CX3CR1 (43) on CD16^+^ monocytes further indicated their involvement in inflammatory immune response. The chemokine receptor CCR5 plays an important role in recruiting these cells into inflamed organs and consumes its own ligands to restrain local chemokine levels, thereby limiting inflammatory cell influx (44). The CCR5 downregulation on CD16^+^ intermediate and non-classical subsets may explain their anti-inflammatory features during the disease course. Both CD16^+^ subsets and CD16^−^ monocytes from SLE patients exhibited a widely changes on cell-surface marker expression, which may be explained by immunosuppressive therapy in patients with SLE (45), but it remains unknown whether treatment with SLE agents can change the monocyte phenotypes and further study was necessary for reasonable explanation in the future.

Disturbed T-cell signaling and Th17/Treg imbalance are documented to play an important role in developing SLE and could be responsible for an increased proinflammatory response, especially in the active form of the disease (4, 46, 47). Human monocytes are well known to influence CD4^+^ cells to differentiate into discrete Th cell subsets. The results presented in this work investigated the effects of each monocyte subset on the control of Th and Treg development in patients with SLE and their healthy counterparts. In contrast to CD16^−^ monocytes, CD16^+^ subset significantly promoted the expansion of IL-4-producing T cells in HCs. Also, CD16^+^ monocyte subset had no effects on Treg induction, supporting its defective inflammation regulatory roles. CD16^+^ subset seemed to be equivalent to CD16^−^ subset at inducing the activation of IFN-γ-producing T cells. Interestingly, Th17 cell development was not influenced by both CD16^+^ inflammatory monocytes and classical CD16^−^ monocytes in HCs. These data suggested the predominant role of circulating CD16^+^ monocyte subset in controlling the balance in Th-polarized immune response. CD16^+^ blood monocytes as well as CD16^−^ subsets can modulate Th1, Th2, and Treg development in patients with SLE. The critical finding of the present study was the identification of each monocyte subset that could modulate Th17 expansion in patients with SLE, to a lesser extent, than in HCs. In recent years, IL-17-producing CD4^+^ T cells have emerged as a major pathogenic T-cell population that is present at increased frequencies in SLE and that correlates with disease severity (47). Recovery of the immune balance between Th17 and regulatory T cells may serve as a treatment for SLE (48). The underlying mechanisms of this expansion of the Th17-cell population are not fully understood, but the present results demonstrated that SLE monocyte subsets were able to drive remarkable Th17-cell expansion *in vitro*, indicating a central role of abnormal monocyte distribution in this process.

Autoantibodies are a hallmark of SLE, and B-cell deletion remains one of the main effective therapies in this disease (49). The present study demonstrated for the first time that both CD16^+^ and CD16^−^ monocytes from patients with SLE exhibited exacerbated capacity for B-cell response, leading to abnormal antibody secretion. In particular, CD16^+^ and CD16^−^ monocytes from patients with SLE could efficiently induce more PBs compared with CD16^+^ and CD16^−^ monocyte subset from HCs. Although HC monocytes were able to induce IgG secretion, CD16^+^, and CD16^−^ monocytes from patients with SLE were more efficient than the corresponding HC monocyte subsets at inducing IgG response. The present results also indicated that the expansion of CD16^+^ monocytes was positively correlated with autoantibody production in patients with SLE, which further confirmed that abnormal monocyte subset was responsible for aberrant B-cell activation. All these suggested that a potential heightened activation state of each monocyte subset in SLE might result in skewed B-cell responses, leading to augment autoantibody production, which was consistent with the finding that B cells were overactivated in SLE (1, 50, 51). Specially, MB activation induced by CD16^+^ monocytes was significantly attenuated, and MB differentiation induced by CD16^−^ monocytes was even inhibited in patients with SLE. The underlying mechanism of this discrepancy need to be further studied.

In summary, the present study demonstrated the expansion of pathogenic CD16^+^ monocytes in SLE, revealing their important roles in stimulating Th cell subsets and the differentiation of B-cell response in diseased condition. These data highlighted the importance of the innate immune system in eliciting pathogenic T- and B-cell responses in SLE and suggested that specific monocyte subsets might be critical targets to control the inflammation.

## Author Contributions

HZ performed most of the experiments. FH, XS, and YS conceived the study and participated in the design and interpretation of results. XZ, XLiu, and XLi helped to collect samples. LZ, LX, LS, and YG participated in the experiments and drafting the manuscript. All the authors read and approved the final manuscript.

## Conflict of Interest Statement

The authors declare that the research was conducted in the absence of any commercial or financial relationships that could be construed as a potential conflict of interest.
