# Supplementary material for: CD16+ Monocyte Subset Was Enriched and Functionally Exacerbated in Driving T-Cell Activation and B-Cell Response in Systemic Lupus Erythematosus
Source: Front Immunol. 2016 Nov 21;7:512. doi: 10.3389/fimmu.2016.00512 (PMC5116853; doi:10.3389/fimmu.2016.00512)
Supplement: Supplementary file 1 [file Presentation_1.PDF]

Supplementary figures and tables.

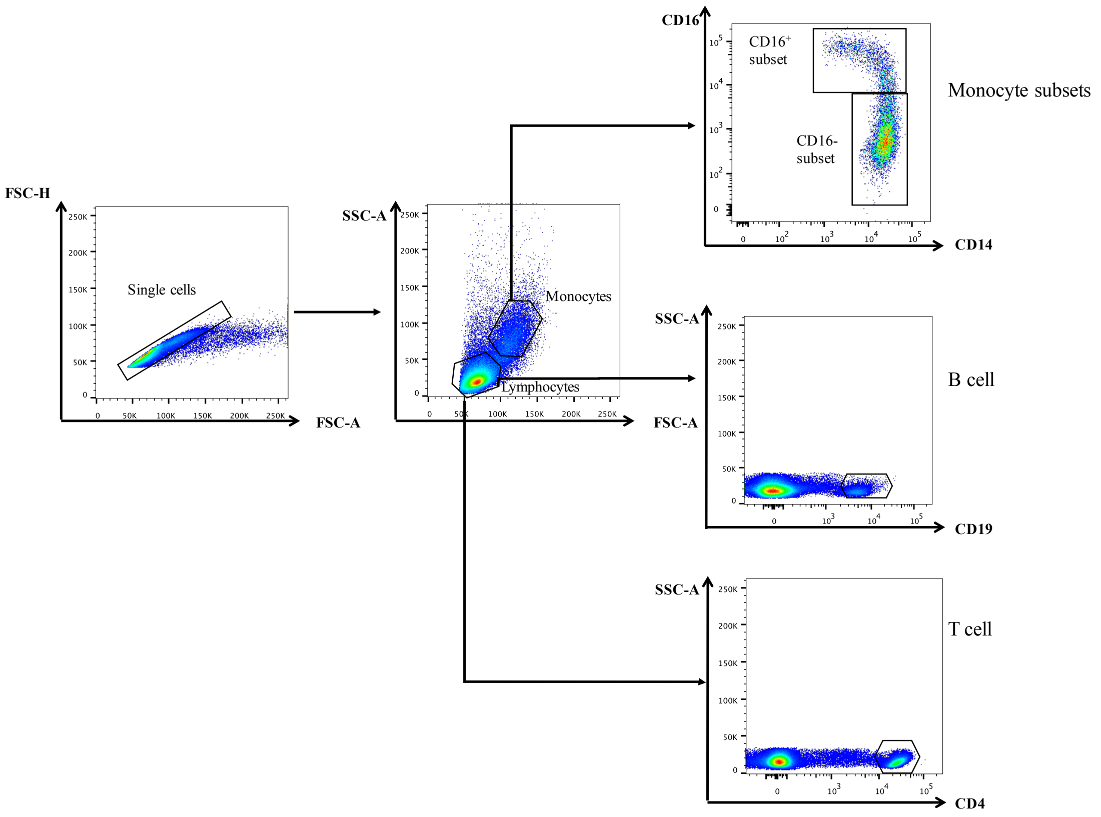

Fig. S1. Monocyte subsets, B cells and T cells was gated based on CD14, CD16, CD19 and CD4 expression.

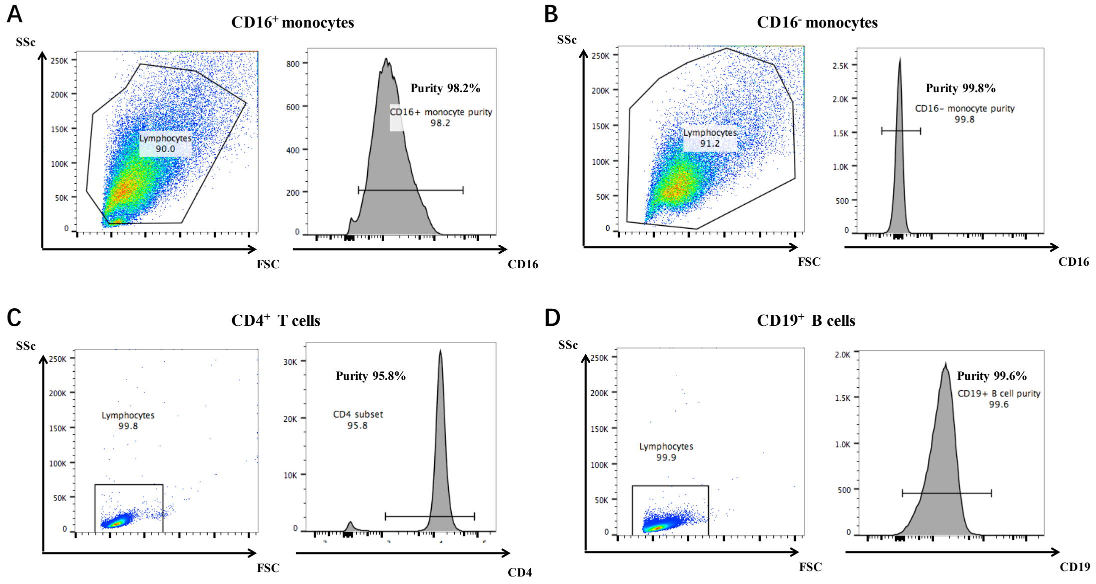

Fig. S2. The purity of sorted CD16^{+} monocytes, CD16^{-} monocytes, CD4^{+} T cell and CD19^{+} B cells were determined by flow cytometry.

Table S1. The frequencies of each monocyte subset in the presence or absence of auto-antibodies in SLE patients.

	AnuA			Anti-Sm Ab			Anti-SSA Ab			Anti-SSB Ab		
	Presence (n=24)	Absence (n=38)	P	Presence (n=9)	Absence (n=53)	P	Presence (n=29)	Absence (n=33)	P	Presence (n=12)	Absence (n=50)	P
$CD16^{+}$ subset (%)	36.93± 18.31	31.75± 14.72	0.701	26.79± 11.56	31.46± 17.93	0.731	32.94± 18.71	34.47± 13.99	0.42	27.81± 19.3	35.21± 15.30	0.067
NCM (%)	16.92± 15.51	13.69± 10.01	0.734	6.73± 2.01	14.44± 13.54	0.053	13.34± 13.60	16.56± 11.36	0.057	11.45± 11.22	15.91± 12.76	0.121
IM (%)	20.01± 10.03	18.06± 9.04	0.757	20.51± 11.47	17.0± 9.11	0.422	19.60± 10.30	17.88 ±8.58	0.359	16.36± 9.45	19.30± 9.33	0.46
CM (%)	61.52± 19.44	67.27± 14.67	0.669	71.39± 11.57	67.27± 18.69	0.797	65.63± 19.62	64.55± 14.07	0.487	71.18± 20.14	63.55± 15.74	0.057

SLE: Systemic Lupus erythematosus; AnuA: antinucleosome antibody; Anti-Sm Ab: anti-Sm antibody; Anti-SSA Ab: anti-SSA antibody; Anti-SSB Ab: anti-SSB antibody; NCM: nonclassical monocytes; IM: intermediate monocytes; CM: classical monocytes. Data were expressed as mean ± SD and analyzed by Mann-Whitney *U* test. P values less than 0.05 were considered significant.

Table S2. The expression of surface markers on $CD16^{+}$ and $CD16^{-}$ monocytes in HCs and patients with SLE.

MFI	CD16+ monocytes			CD16- monocytes		
	HCs	SLE patients	P	HCs	SLE patients	P
CD80	291±223	328±162	0.112	227±146	196±87	0.667
CD86	468±284	483±230	0.750	279±254	335±163	0.195
HLA-DR	15011±12265	9470±7921	0.042*	9760±8047	4477±3124	< 0.001***
CD163	1574±1049	9935±8301	< 0.001***	1573±1270	7682±6013	< 0.001***
CCR5	993±587	1622±449	< 0.001***	1253±197	1843±237	0.927
CX3CR1	5435±2794	4607±2475	0.146	1799±1104	2951±1250	< 0.01**

SLE: Systemic Lupus erythematosus; MFI: Median fluorescence intensity. Data were expressed as mean ± SD and analyzed by Mann-Whitney *U* test. P values less than 0.05 were considered significant. **P < 0.05, **P < 0.01, ***P < 0.001.*
